# Carbapenemase-Producing Extraintestinal Pathogenic *Escherichia coli* From Argentina: Clonal Diversity and Predominance of Hyperepidemic Clones CC10 and CC131

**DOI:** 10.3389/fmicb.2022.830209

**Published:** 2022-03-18

**Authors:** María Belén Sanz, Denise De Belder, JM de Mendieta, Diego Faccone, Tomás Poklepovich, Celeste Lucero, Melina Rapoport, Josefina Campos, Ezequiel Tuduri, Mathew O. Saavedra, Claudia Van der Ploeg, Ariel Rogé, Carbapenemases-ExPEC Group, A. Piersigulli, Fernando Pasteran, Alejandra Corso, Adriana E. Rosato, Sonia A. Gomez

**Affiliations:** Sanat. Privado aconcagua (CDBA), Sanat. Anchorena, Htal. Britanico, Hzga. Carlos Bocalandro, Clinica Modelo Moron, Clinica Y. Maternidad Suizo Argentina, de Agudos Cosme Argerich, “C. G. Durand”, H. Evita de Lanus, ICCYC, Fundacion Favaloro, H. de Niños Ricardo Gutierrez, H. Area Cipolletti, H. de Agudos Parmenio Piñero, H. Sanguinetti (Comodoro Rivadavia), H. “Julio Perrando”, H. Vlla. El Libertador Ppe. de Asturias (Córdoba), H. Privado de Cordoba, H. Regional Rio Gallegos; H. San Martin (Parana), H. San Roque (CDBA); H. Infantil Municipal de Cordoba, H. Italiano (CABA), H. Gral de Agudos “J. A. Fernandez”, Laboratorio San Carlos, Laboratorio Virreyes, Instituto de Investigaciones Médicas Alfredo Lanari, Da.; H. Guillermo Rawson (San Juan), H. Militar Central Cortez B.; Sanatorio Mitre, Bottinelli, Cifarelli; H. Prov. de Neuq. “Castro Rendón”, Htall Arturo Oñativia - Rafael Calzada, B. S. A. S., H. Gral de Agudos “J. A. Penna” (CABA), H. Nac. Alejandro Posadas, H. de Agudos Ramos Mejia, H. Guillermo Rawson (Cordoba), Sanatorio Trinidad Mitre, Sanatorio Güemes (Caba), HIGA José San Martín de La Plata, H. Velez Sarsfield; ^1^Servicio Antimicrobianos, Laboratorio Nacional de Referencia en Resistencia a los Antimicrobianos (LNRRA), INEI-ANLIS “Dr. Carlos G. Malbrán”, Buenos Aires, Argentina; ^2^Plataforma Genómica y Bioinformática (PLABIO), INEI-ANLIS “Dr. Carlos G. Malbrán”, Buenos Aires, Argentina; ^3^Consejo Nacional de Investigaciones Científicas y Técnicas (CONICET), Buenos Aires, Argentina; ^4^Department of Pathology and Genomic Medicine, Center for Molecular and Translational Human Infectious Diseases Research, Houston Methodist Hospital, Houston Methodist Research Institute, Houston, TX, United States; ^5^Servicio de Antígenos y Antisueros, INPB-ANLIS “Dr. Carlos G. Malbrán”, Buenos Aires, Argentina; ^6^Department of Pathology and Molecular Microbiology Diagnostics-Research, Riverside University Health System, Moreno Valley, CA, United States; ^7^School of Medicine, University of California, Riverside, Riverside, CA, United States

**Keywords:** ExPEC, carbapenemase, *Escherichia coli*, phylogeny, high risk clone

## Abstract

Extraintestinal pathogenic *Escherichia coli* (ExPEC) causes infections outside the intestine. Particular ExPEC clones, such as clonal complex (CC)/sequence type (ST)131, have been known to sequentially accumulate antimicrobial resistance that starts with chromosomal mutations against fluoroquinolones, followed with the acquisition of *bla*_CTX–M–15_ and, more recently, carbapenemases. Here we aimed to investigate the distribution of global epidemic clones of carbapenemase-producing ExPEC from Argentina in representative clinical isolates recovered between July 2008 and March 2017. Carbapenemase-producing ExPEC (*n* = 160) were referred to the Argentinean reference laboratory. Of these, 71 were selected for genome sequencing. Phenotypic and microbiological studies confirmed the presence of carbapenemases confirmed as KPC-2 (*n* = 52), NDM-1 (*n* = 16), IMP-8 (*n* = 2), and VIM-1 (*n* = 1) producers. The isolates had been recovered mainly from urine, blood, and abdominal fluids among others, and some were from screening samples. After analyzing the virulence gene content, 76% of the isolates were considered ExPEC, although non-ExPEC isolates were also obtained from extraintestinal sites. Pan-genome phylogeny and clonal analysis showed great clonal diversity, although the first phylogroup in abundance was phylogroup A, harboring CC10 isolates, followed by phylogroup B2 with CC/ST131, mostly H30Rx, the subclone co-producing CTX-M-15. Phylogroups D, B1, C, F, and E were also detected with fewer strains. CC10 and CC/ST131 were found throughout the country. In addition, CC10 nucleated most metalloenzymes, such as NDM-1. Other relevant international clones were identified, such as CC/ST38, CC155, CC14/ST1193, and CC23. Two isolates co-produced KPC-2 and OXA-163 or OXA-439, a point mutation variant of OXA-163, and three isolates co-produced MCR-1 among other resistance genes. To conclude, in this work, we described the molecular epidemiology of carbapenemase-producing ExPEC in Argentina. Further studies are necessary to determine the plasmid families disseminating carbapenemases in ExPEC in this region.

## Introduction

Antimicrobial resistance due to carbapenemases has settled in the last 20 years as one of the main problems for the treatment of hospital-acquired infections globally (Stoesser et al., 2017). These enzymes bear a broad-spectrum hydrolytic activity toward beta-lactam antibiotics, including last-resort drugs like carbapenems (Doi and Paterson, 2015). *Klebsiella pneumoniae* carbapenemase (KPC) and New Delhi metallo-β-lactamase (NDM) carbapenemases represent two of the five most relevant carbapenemase enzymes, the others being the metallo-ß-lactamases VIM and IMP and the oxacillinase OXA-48-like (Stoesser et al., 2017). Carbapenemase genes are altogether known to disseminate from diverse *Enterobacterales* on conjugative or mobile plasmids that harbor additional resistance genes to multiple antimicrobial families (Doi and Paterson, 2015), like aminoglycosides, quinolones, sulfonamides, macrolides, and phenicols among others, resulting in limited therapeutic options for the patients. In addition, carbapenemases are located in mobile genetic elements, such as the recognized Tn*4401* that harbors *bla*_*KPC*_ (Gomez et al., 2011) or the multiple-variant derivatives of Tn*125* that harbor *bla*_*NDM*_ (Martino et al., 2019). For these reasons, continuous surveillance of carbapenem-resistant *Enterobacterales* is necessary to monitor the emergence and dissemination of these microorganisms.

*Escherichia coli* is a commensal bacterium in mammals, and although most strains are harmless, others are relevant infectious agents and a major cause of hospital- and community-acquired infections (Pitout, 2012b). In particular, *E. coli* can be grouped into eight phylogroups termed A, B1, B2, C, D, E, F, and G (Johnston et al., 2021), and the assignment of a particular strain to a phylogroup reveals a strain’s ecological niche, lifestyle, and propensity to cause a disease (Beghain et al., 2018). In this sense, epidemiological studies require reliable identification of the genetic background of clinically relevant bacteria achieved by whole-genome sequencing, phylogeny studies, and multilocus sequence typing (MLST; Beghain et al., 2018).

*E. coli* displays a peculiar genome plasticity that enables the flow of genes in and out of the cell, generating high variability of the gene content among lineages that include resistance and virulence genes (Gomi et al., 2017; Horesh et al., 2021). Within this species, extraintestinal pathogenic *E. coli* (ExPEC) is a versatile pathotype that typically causes urinary tract infections, pyelonephritis, sepsis, pneumonia, and meningitis (Bok et al., 2020). ExPEC strains generally occupy a niche in the gut microbiota of humans and other animals, and it is from this reservoir that they spread to cause extra-intestinal infections (Manges et al., 2019). Moreover, ExPEC possesses specialized virulence factors that facilitate the bacteria–host interaction to cause invasion, colonization of diverse hosts, and evasion of the immune system and to induce disease outside the gastrointestinal tract (Tan et al., 2012). Distinctive ExPEC virulence factors include adhesins (type I and P fimbriae), iron acquisition and utilization systems (aerobactin and salmochelin siderophores), protectins (structural components of the bacterial outer membrane), toxins (hemolysin, cytotoxic necrosis factor), and biofilm formation factor (antigen 43) (Bok et al., 2020).

Global studies have shown that the most relevant ExPEC clone is ST131 or clonal complex (CC)131 within phylogroup B2 (Pitout and DeVinney, 2017). The evolution of this clone demonstrated, first, the sequential resistance accumulation of *gyrA* and *parC* mutations conferring fluoroquinolone resistance and, second, the acquisition of a plasmid harboring *bla*_CTX–M–15_, an extended-spectrum beta-lactamase conferring resistance to 3rd-generation cephalosporins (Pitout and DeVinney, 2017). This clone was disseminated worldwide, and soon enough it was reported as co-producing KPC- or NDM-type carbapenemases, limiting treatment options even more (Pitout and DeVinney, 2017; Stoesser et al., 2017).

CC10 (ST10 and related STs) belongs to phylogroup D and is recognized today as an emerging clone of ExPEC. CC10 has been detected in a broader range of niches that include the clinical setting, food animals, and the environment (Manges et al., 2019). It has also been associated with resistance to fluoroquinolones and β-lactams and has been distinguished as a putative reservoir of the transferable colistin resistance gene *mcr-1* (Massella et al., 2021; Skarżyńska et al., 2021).

In a previous study (De Belder et al., 2018), in the context of carbapenem-resistant *Enterobacterales*, surveillance was carried out by the National Reference Laboratory in Antimicrobial Resistance (NRLAR) in Argentina among 29 KPC-producing *E. coli*, five of which belonged to ST131. In consequence, we aimed to investigate the distribution of global epidemic clones of carbapenemase-producing ExPEC across Argentina. To accomplish this purpose, we performed a longitudinal and retrospective study on 71 carbapenemase-producing *E. coli* isolates received at the NRLAR for molecular characterization between July 2008 and March 2017.

## Materials and Methods

### Isolate Collection, Susceptibility Testing, and PCR

Between July 2008 and March 2017, 974 clinical *E. coli* isolates were reported by WHONET-Argentina network (89 laboratories) as resistant to at least one carbapenem. During that time period, 160 isolates suspected of carbapenemase production were referred to the NRLAR by health institutions throughout the country for further study. These 160 *E. coli* isolates were defined by conventional biochemical methods, such as negative oxidase disk, positive indole production, negative citrate utilization, and MaldiTof MS. All the isolates were confirmed to produce carbapenemase by phenotypic and microbiological methods: ertapenem inhibition zone ≤ 22 mm, positive Triton-Hodge Test, positive Blue Carba Test, and positive synergy between a carbapenem disk and amino-phenyl boronic acid disk or EDTA disk for the detection of KPC or metallo-β-lactamases, respectively. The phenotypic determination protocols and interpretation criteria were those of the Clinical and Laboratory Standards Institute (CLSI, 2019). Of these, 71/160 representative carbapenemase-producing *E. coli* isolates were selected for further molecular study, contemplating one isolate per patient referred from all geographic regions, institutions, and years of isolation and excluding isolates that may be part of an outbreak.

Susceptibility to relevant antimicrobials was determined by disk diffusion following the CLSI guidelines [M100 CLSI (2019)]. Susceptibility to colistin was determined by broth microdilution minimum inhibitory concentration (MIC). Colistin and fosfomycin breakpoints were those defined by EUCAST, while tigecycline breakpoint was by FDA. Reduced susceptibility category included resistant (R) plus intermediate (R + I) isolates. All carbapenemase genes and alleles were preliminarily confirmed by PCR using detection primers, as described (De Belder et al., 2018), and Sanger sequencing.

The strains were also serotyped by standard procedures using specific rabbit antisera against *E. coli* somatic (O) antigen and the flagellar (H) antigen prepared by immunization of rabbits as previously described (Ørskov and Ørskov, 1984).

### Whole-Genome Sequencing

DNA was extracted with QIACube DNAMini Kit (Qiagen, Hilden, Germany), and sequencing was performed using the Nextera XT DNA library preparation kit. The extracted DNA was sequenced using Illumina’s HiSeq2000 instrument at the Epigenetics and Genomic Laboratory at Weill Cornell Medical College, NY, United States, to generate 150-bp paired-end reads. The isolates whose quality was low were resequenced by Illumina MiSeq to generate 250-bp paired-end reads using the same DNA extraction procedure at the Genomic and Bioinformatic Platform, INEI-ANLIS “Dr. Carlos G. Malbrán”.

### Genome Assembly, Annotation, and Analysis

Paired-end reads were trimmed with Trim Galore (V.0.6.3) and analyzed for quality with FASTQC (V.0.11.5) (Wingett and Andrews, 2018). Kraken2 (V.2.0.7-beta) was used to confirm the species (Wood and Salzberg, 2014). Reads were *de novo* assembled with SPAdes (3.13.0), and the quality was evaluated with QUAST (V.5.0.2) (Supplementary Table 1; Gurevich et al., 2013). The annotation of the genomes was done with Prokka (V.1.14.0) (Ørskov and Ørskov, 1984). ARIBA (Hunt et al., 2017) was run to determine resistance genes (ResFinder, V.2.14.4) and virulence genes (VFDB_core V.214.4). Furthermore, the alleles of the resistance genes were confirmed using the assemblies *in silico* and AMRFinder (V.3.8.4) or by conventional Sanger sequencing when required. *In silico* serotyping was done with SRST2_serotypes using the EcOH database (Ingle et al., 2016) and confirmed by serology. The analysis of *bla*_*KPC*_ close genetic environment was done with TetTyper (Sheppard et al., 2018). To confirm ExPEC isolates, we analyzed the virulence gene content if positive for ≥ 2 of *papAH* and/or *papC* (P fimbriae), *sfa/focDE* (S and F1C fimbriae), *afa/draBC* (Dr-binding adhesins), *iutA* (aerobactin siderophore system), or *kpsM* II (group 2 capsules) (Tetzschner et al., 2020). The sequence type (ST) for each genome was determined by running ARIBA sequence type (MLST, V.2.14.6), and clonal complexes (CC) were obtained by submitting trimmed reads to Enterobase (Zhou et al., 2020). Both methods determine *E. coli* MLST according to Achtman scheme from PubMLST database on November 1, 2021. By definition, the CC nucleates the ST that most likely represents the founding genotype or primary founder. Here the CCs of related STs were grouped according to a default group definition of sharing at least five of seven alleles. The *fimH* gene, encoding the type 1 fimbrial adhesin, was characterized using *fim*Typer which was from the Center of Genomic Epidemiology (Roer et al., 2017). In our analysis, fluoroquinolone-resistant ST131-H30 isolates were classified as H30Rx when also producing the ESBL. In turn, H30R isolates susceptible to cephalosporins or non-CTX-M-15-producers were classified as H30R1 (Johnson et al., 2017).

### Phylogenetic Analysis

Phylogenetic analysis was performed with the WGS dataset of reference strains belonging to 8 phylogroups (A, B, B2, C, D, E, F, and G) and the 71 ExPEC sequenced. The complete genomes of the reference strains were downloaded from NCBI and annotated with Prokka to achieve uniformity within the dataset. Roary was used to infer the core-gene phylogeny from the core-gene alignment (concatenated genes present in >99% of the genomes with >95% of nucleotide identity) generated for all the isolates with the annotated assemblies (Page et al., 2015). This core-genome alignment was used to generate a single-nucleotide polymorphism (SNP) alignment with SNP sites (V.2.3.3) (Page et al., 2016). A maximum likelihood tree was constructed using RAxML (V.8.2.11) (Stamatakis, 2014) under the GTR model with the gamma distribution to model site heterogeneity (GTRGAMMA) using 1,000 bootstrap replicates. Fastbaps was used to cluster sequences in the *E. coli* phylogeny, which provides Bayesian hierarchical clustering of the data (Tonkin-Hill et al., 2019). The alignments obtained to build the tree were used as the input for Fastbaps. The phylogroup assignment was performed *in silico* according to the Clermont PCR method using the command line tool (Shaik et al., 2017). The interactive tree with the geographic location of the isolates was loaded into Microreact (Argimon et al., 2016). The tree was midpointed and visualized with iTOL (V.6.5).

### Data Availability

The sequenced data have been deposited in GenBank under bioproject accession PRJNA784589.

## Results

### Epidemiological and Phenotypic Analysis of the Clinical Isolates

**FIGURE 1:**
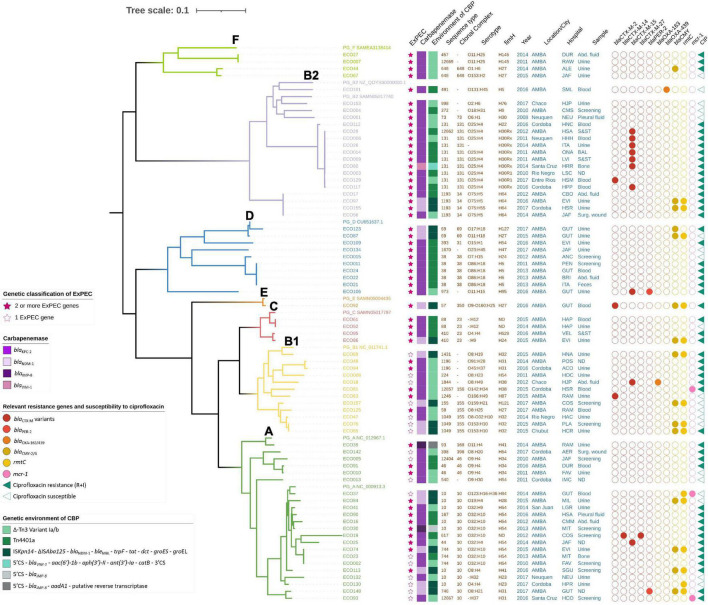
Maximum likelihood tree of 71 *Escherichia coli* isolates belonging to phylogroups A, B1, B2, C, D, E, and F with metadata. The tree was drawn to scale, with branch lengths measured in numbers of substitutions per site. The dataset contained 1,061 core genes and 68,434 SNPs. Abd., abdominal; CIP, ciprofloxacin; CBP, carbapenemase.

Carbapenem-resistant *E. coli* were obtained from nine provinces across the country and 46 health centers (Figure 1 and Microreact map in https://microreact.org/project/1YdHkLWgkKvWXchJ2hrEs3-ecoscbpargentinamidpoint). The majority of the isolates were referred from the two largest locations in Argentina: AMBA (City of Buenos Aires and surroundings) [no. of isolates/total no. of isolates—%; (51/71, 72%)] followed by the second largest city of Córdoba (8/71, 11%) (Table 1). The rest of the provinces referred 1 to 3 isolates each.

**TABLE 1:** Epidemiological data of the isolates.

		Number of isolates (%)
		*n* = 71
Location/city	AMBA	51 (72)
	Córdoba	8 (11)
	Neuquén	3 (4)
	Chaco	2 (3)
	Santa Cruz	2 (3)
	Río Negro	2 (3)
	Entre Ríos	1 (1)
	Chubut	1 (1)
	San Juan	1 (1)
Sample	Urine	27 (38)
	Blood	12 (17)
	Screening	11 (15)
	Abdominal fluid	5 (7)
	S&ST	3 (4)
	Surgical wound	2 (3)
	Pleural fluid	2 (3)
	Bone	2 (3)
	Feces	1 (1)
	BAL	1 (1)
	ND	5 (7)
Infection	Invasive^a^	52 (73)
	Non-invasive	19 (27)
Sex	Female	30 (42)
	Male	39 (55)
	nd	5 (3)
Age	Median	62 years
	Range	1 month–94 years
Carbapenemase	*bla*_*KPC–2*_	52 (73)
	*bla*_*NDM–1*_	16 (23)
	*bla*_*IMP–8*_	2 (3)
	*bla*_*VIM–1*_	1 (1)
Other resistance genes	*bla*_CTX–M_	15 (22)
	*bla*_*PER–2*_	2 (3)
	*bla*_*CMY*_	17 (24)
	*bla*_*OXA–163*_ ^b^	1 (1)
	*bla*_*OXA–43*_ ^b^	1 (1)
	*mcr-1*	3 (4)
	*rmtC* ^b^	15 (21)

*S&ST, skin and soft tissue; BAL, brochoalveolar lavage; nd, not determined; AMBA, Metropolitan Area of Buenos Aires, including the capital district and surroundings.*

*^a^Considering invasive infections, samples from urine, blood, S&T, pleural fluid, bone, abdominal fluids, and BAL.*

*^b^Detected by whole-genome sequencing.*

**FIGURE 2:**
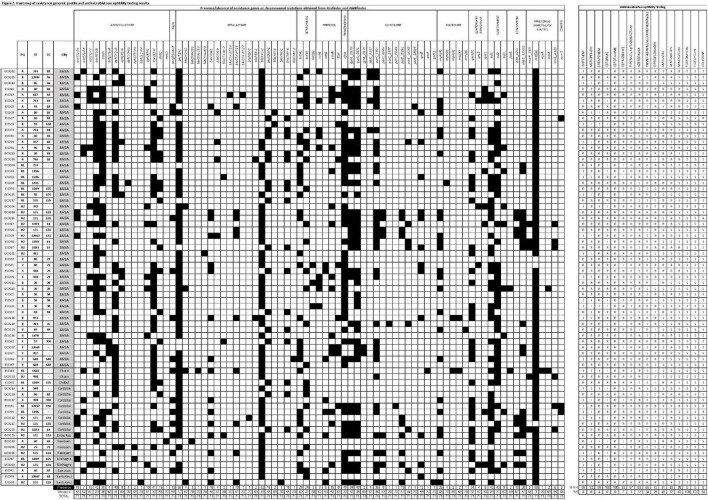
Heat map of the resistance genomic profile and antimicrobial susceptibility testing results. The figure shows the presence and the absence of resistance genes or chromosomal mutations represented by black and white squares, respectively. The isolates were organized by geographic location. Column totals and percentages can be seen at the bottom of the figure. PG, phylogroup; ST, sequence type; CC, clonal complex; AG, aminoglycosides; Q, quinolone; AMP, ampicillin; CHL, chloramphenicol; RIF, rifampin; TET, tetracycline; R, resistant; I, intermediate; S, susceptible.

The median age was 62 years old, but the age range was very broad, including a newly born baby (1 month old—94 years) (Table 1). In total, 55% of the isolates were obtained from males. In addition, the isolates were mostly recovered from different sources of invasive infections (52/71, 73%), including urine (27/71, 38%), blood (12/71, 17%), abdominal fluid (5/71, 7%), skin and soft tissue (3/71, 4%), bones (2/71, 3%), pleural fluid (2/71, 3%) and bronchoalveolar lavage (1/71, 1%) (Table 1). The rest of the non-invasive isolates were obtained from screening (11/71, 15%) and other sources, such as feces or surgical wound (8/71, 11%) (Table 1). In all isolates, carbapenemase genes were confirmed, resulting on *bla*_KPC–2_ in 52/71 (73%), *bla*_*NDM–*1_ in 16/71 (23%), *bla*_*IMP–*8_ in 2/71 (3%), and *bla*_*VIM–*1_ in 1/71 (1%). Other additional epidemiologically relevant mechanisms were detected as follows: *mcr-1* (3/71, 4%), *rmt*C (15/71, 21%), *bla*_*PER–*2_ (2/71, 3%), *bla*_CTX–M_ (15/71, 22%) disclosed as 10 *bla*_CTX–M–15_, 3 *bla*_CTX–M–2_, 1 *bla*_CTX–M–14_, and 1 *bla*_CTX–M–27_ (Table 1 and Figures 1, 2).

The overall susceptibility testing results showed 100% resistance to cefotaxime and cefepime, and 93% showed reduced susceptibility [includes resistant plus intermediate (R + I) categories] to ceftazidime. A high proportion of resistance to carbapenems was found, detecting 96% R + I to ertapenem and imipenem and 92% to meropenem. Moreover, 97% of the isolates showed reduced susceptibility to piperacillin/tazobactam and 83% to aztreonam. The level of resistance toward other antimicrobial families was variable: 63% of the strains were R + I to gentamicin and 51% to amikacin, and 72% of the strains were R + I to ciprofloxacin and 66% to trimethoprim/sulfamethoxazole (Figure 2). Among the antibiotics that are usually therapeutic alternatives for multidrug-resistant microorganisms, we found that minocycline displayed 38% R + I, while all the strains were susceptible to fosfomycin and tigecycline (Figure 2). Resistance to colistin was tested by broth microdilution. The MIC results showed that 4 isolates were resistant with MICs ≥ 4 μg/ml. *mcr-1* was confirmed by PCR in 3 of the 4 resistant isolates (Figure 2).

### Molecular Analysis, Phylogeny, and Clonal Distribution of Extraintestinal Pathogenic *Escherichia coli*

A core gene alignment of 913,996 bases was produced from 1,061 core genes (≥99%), yielding a final alignment of 68,434 SNP sites. The phylogenetic analysis of the isolates showed that they belonged to seven of the known phylogroups distributed as follows: 31% (22/71) belonged to phylogroup A, 25.4% (18/71) to phylogroup B2, 16.9% (12/71) to phylogroup B1, 14.1% (10/71) to phylogroup D, 5.6% (4/71) to phylogroup C, 5.6% (4/71) to phylogroup F, and 1.4% (1/71) to phylogroup E (Figure 1 and Microreact: https://microreact.org/project/uNGRc9b3gvT6jApXgZBToA/4c 5d3f1d). Phylogroup G was not detected among the isolates.

Total resistance gene content and virulence gene content can be found in Figure 2 and Supplementary Table 2, respectively. The analysis of virulence gene content resulted in 54 of 71 (76%) isolates that fulfilled the criteria of ExPEC (Table 2 and Figure 1; Tetzschner et al., 2020). In addition, 68 of 71 (96%) isolates harbored type 1 fimbriae *fimH*. The capsule *kps*M was found in 39% of the isolates, while genes coding for iron uptake (*e*.*g*., *chuA, fyuA*, *irp*, ituA, and *iroN*) were found in approximately 50% of the isolates. In addition, the median score was four virulence genes per strain. The median virulence scores for each phylogroup were 9 for D, 8 for B2, 4 for F, 3 for C, 2 for A, and 1 for B1 (Table 2).

**TABLE 2:** Virulence gene content per phylogroup, median, and total scores.

	Genes	Phylogroup								
		A (*n* = 22)	B2 (*n* = 18)	D (*n* = 10)	B1 (*n* = 12)	C (*n* = 4)	F (*n* = 4)	E (*n* = 1)	Total (*n* = 71)	Total%
Adhesin	dra	1	1	4	0	0	0	0	6	8
	Fim	21	18	10	12	2	4	1	68	96
	nfaE	0	0	0	0	0	0	0	0	0
	Pap	1	4	4	1	0	2	0	12	17
	Sfa	0	3	0	0	0	0	0	3	4
	yfcV	0	0	0	0	0	0	0	0	0
	focC	0	3	0	0	0	0	0	3	4
Protectin	Iss	0	0	0	0	0	0	0	0	0
	kfiC	0	0	0	0	0	0	0	0	0
	Tra	0	0	0	0	0	0	0	0	0
	kpsM	2	13	9	0	0	4	0	28	39
Siderophore	Chu	0	18	10	0	0	4	1	33	46
	fyuA	5	18	7	1	2	2	1	36	51
	Iha	0	0	0	0	0	0	0	0	0
	Irp	5	17	7	1	2	2	1	35	49
	iuc	10	14	7	4	3	1	1	40	56
	iutA	11	14	7	4	4	1	1	42	59
	iroN	6	3	0	4	2	0	1	16	23
Toxin	sat	1	10	7	0	0	0	0	18	25
	hlyD	0	5	1	0	0	0	0	6	8
	cnf1	0	4	1	0	0	0	0	5	7
	tsh	0	0	0	0	0	0	0	0	0
Miscellaneous	malX	0	0	0	0	0	0	0	0	0
	ompT	0	0	0	0	0	0	0	0	0
	cvaC	0	0	0	0	0	0	0	0	0
	usp	0	0	0	0	0	0	0	0	0

Score (number of genes)		63	145	74	27	15	20	7		

Median		2	8	9	1	3	4	-		

The phylogenetic analysis of phylogroup A showed a scattered distribution of branches (Figure 1). This phylogroup was composed of 22/71 (31%) isolates expressing KPC-2 (14/22, 64%), NDM-1 (6/22, 26%), and IMP-8 (2/22, 10%) recovered from 19 institutions, mainly from AMBA, of which 12/22 (54%) were ExPEC. In particular, CC10 was represented by 16 isolates (22.5%), making this CC the major clone detected (Figure 1). Within this CC, eight isolates belonged to ST10, and others were single locus variants like ST44 (*n* = 1), ST744 (*n* = 3), and ST167 (*n* = 1) and double locus variants ST746 (*n* = 1) and ST617 (*n* = 1). One isolate belonged to ST12667 as a satellite CC10. The CC10 isolates (16/22, 73%) were KPC-2 (9/16, 56%), NDM-1 (6/16, 38%), and IMP-8 (1/16, 6%) producers. Interestingly, two CC10 isolates, ECO37 (ST10, NDM-1) and ECO93 (ST12667, KPC-2), coproduced MCR-1. In addition, ECO148 (NDM-1) was co-producer of PER-2. In particular, seven isolates expressed O32:H10 and 10 the *fim*-type H54. ECO19 (ST617/CC10, O32:H10, untypable *fim* type) was a coproducer of KPC plus CTX-M-14 and CTX-M-27. Three isolates (ST744) stand together on a branch, one ExPEC-NDM-1/CMY-6 and 2 KPC-2 producers, obtained in different years and hospitals.

Phylogroup B2 was composed of 18/71 (25%) ExPEC isolates (Figure 1) expressing mostly KPC-2 and also VIM-1 and NDM-1. These isolates belonged to 7 STs and 3 CCs (Figure 1). The sub-tree that holds phylogroup B2 shows three main clades. One displays phylogenetic closeness holding CC131 isolates (10/18, 55%) that were obtained between 2011 and 2017 mainly from infections in 10 hospitals from different cities across the country. Of these, nine were KPC-2 producers, and one was VIM-1 producer. Seven were co-producers of *bla*_CTX–M–15_ (*fim*H30Rx). ECO112, a *fim*H22-KPC-2 isolate, was susceptible to fluorquinolones and harbored the pAMP-C *bla*_*FOX–*5_. Resistance to fluorquinolones in CC131 was due to chromosomal *gyrA/parC/parE* substitutions with or without *qnr* (Figures 1, 2). Two isolates were H30R1, of which ECO129 harbored *bla*_CTX–M–2_. Interestingly, within ST131, ECO14 showed phenotypic resistance to colistin (MIC ≥ 4 μg/ml) but was negative for *mcr* either by PCR or by *in silico* resistance gene search (Resfinder, Amrfinder, and manually). Therefore, we searched for chromosomal mutations and found six mutations in *pmr*B (H2R, E123D, R155Q, D283G, V351I, and S202P) and 1 mutation in *pmr*A (T31S) that may be associated with the colistin resistance observed in that strain, considering that some mutations have already been reported (Aghapour et al., 2019). Another clade holds six isolates with distant branching detected between 2008 and 2017 in three cities. Interestingly, ECO101 (ST491, O131:H45, *fim*H5) was distinctive as KPC-2 and OXA-439 producer. OXA-439 differs from the locally disseminated OXA-163 in a point mutation of tyrosine for histidine (Y123H). Finally, a third branch held 4 isolates that belonged to CC14 (ST1193), O75:H5, *fim*H64 that clustered together phylogenetically and appeared well separated from other STs within phylogroup B2. Two of these were NDM-1/CMY-6 producers, and 2 were KPC-2 producers. They did not have an epidemiological link between them. Phylogroup D was composed of 10/71 (14%) ExPEC isolates, of which five were obtained from urine samples. This phylogroup was represented in five STs and three CC. All isolates were recovered from AMBA in seven hospitals between 2011 and 2017 (Figure 1). Moreover, the resistance to fluorquinolones was variable. The phylogenetic analysis of the sub-tree distinguishes a homogeneous branch holding five isolates of CC38 (ST38), four expressing O86:H18 serotype and three *fim*H5 obtained from diverse sources and in different years, indicating no epidemiological link between them. A distinct isolate was ECO106 (ST973, O11:H15, *fim*H47), a KPC-2, CTX-M-15, PER-2, and *qnr*B1 producer, among other resistance genes (Figure 2), that stands alone in a monophyletic branch. Other findings within this phylogroup were two CC69 (ST69) NDM-1/CMY-6 producers, expressing the flagellar antigen H18, obtained in the same hospital 2 years apart.

Phylogroup B1 was composed of 12/71 (17%) isolates obtained between 2011 and 2017 from 5 cities around the country and 11 hospitals (Figure 1). This phylogroup was composed of 8 (66%) KPC-2 producers, of which 1 co-produced MCR-1 (ECO81) and 1 co-produced OXA-163 (ECO18) (Figure 2), and 4 (34%) NDM-1/CMY-6 were producers. Only 5 of 12 (42%) ExPEC according to the virulence gene content criteria, even though non-ExPEC isolates were obtained from infections of extra-intestinal sites. The sub-tree (Figure 1) reflects heterogeneity in the distribution of branches nucleated in nine STs, two CCs, 11 serotype combinations, and seven *fim*H types. In particular, five isolates belonged to CC155, of which three were ST1049/*fim*H32 isolated in different hospitals. Finally, two ExPEC isolates (ECO49 and ECO94) belonged to ST1196 (*fim*H31) isolated 2 years apart in different provinces. Fluorquinolone resistance or reduced susceptibility was seen in eight isolates of diverse STs.

Other known *E. coli* phylogroups (C, E, and F) were represented by nine ExPEC isolates obtained in AMBA between 2011 and 2016 (Figure 1). Phylogroup C was represented by four isolates of CC23, where two KPC-2 belonged to ST88, with undetermined O antigen, H12 flagellar antigen and undetermined *fim*H type. The other two strains within this phylogroup belonged to ST410. Phylogroup F was represented also by four KPC-2 producers, isolated between 2011 and 2015 in four different hospitals. Of these, ECO44 and ECO67 belonged to CC648 (ST648) expressing different serotypes (O1:H6 and O153:H2, respectively) but the same *fim*H27. ECO44 also harbored *bla*_*CMY–*2_. The other two isolates, ECO7 and ECO27, expressed the same serotype and *fim* type, O11:H25 and *fim*H145, respectively. Finally, phylogroup E was represented by ECO92 (O9/O160:H25, *fim*H27) isolated in 2016. This isolate was an NDM-1, CTX-M-2, and CMY-6 producer among other genes (Figure 2) and belonged to CC350 (ST57).

When analyzing the evolution of genes and clones, we could observe that the diversity of clones increased over the years (Table 3). CC73 (in phylogroup B2) was the first KPC-producing *E. coli* detected in Argentina in 2008. That clone was not detected again over the 9-year span. In contrast, predominant clones such as CC10 (in phylogroup A) and CC131 (in phylogroup B2) were detected since 2010. In 2014, NDM was first detected in an isolate that belonged to CC10, and as a consequence of the incorporation of NDM in the local epidemiology, the frequency of gene detection and diversity of clones increased since then (Table 3).

**TABLE 3:** Frequency of clonal complexes over the years.

Year	2008	2010	2011	2012	2013	2014	2015	2016	2017	Total
**Clonal complex**										
73	1									1
10		1		2	2	3	2	3	3	16
131		1	3	1		2		2	1	10
46		1	1					1		3
Other CC/ST		1	3	1		2	2	3	2	14
38			1	1	3			1		6
14				1		1		1	1	4
23						1	2	1		4
155						1	3		2	6
648						1	1			2
168						1				1
69							1		1	2
350								1		1
398									1	1

Total	1	4	8	6	5	12	11	13	11	71

Frequency	1	4	4	5	2	8	6	8	7	

When looking at the serotypes, the O25:H4 combination was expressed in 9/71 (12.7%) in phylogroup B2, followed by O32:H10 (7/71, 9.9%) in phylogroup A, O86:H18 (4/71, 5.6%) in phylogroup D, and O75:H5 in phylogroup B2 (3/71, 4.2%) (Figure 1). The distribution of *fim* types can be seen in Figure 1. H54 was the most abundant (12/71, 17%), followed by H30Rx (7/71, 10%). Others were H5, H27, H31, H32, and H64 represented by five and four isolates.

### Genetic Environment of the Carbapenemase Genes

The genetic environment of *bla*_KPC–2_ was studied considering that, in the local epidemiology, Tn*4401a* was widely disseminated in KPC-producing *K. pneumoniae* (ST258) and the ΔTn*3*-Variants 1a/b in other *Enterobacterales* (De Belder et al., 2018). When we analyzed the 52 KPC-producing *E. coli*, we found that 25 isolates (48%) harbored *bla*_KPC–2_ in Tn*4401a* (Figure 1), while the remaining 52% of the isolates harbored *bla*_*KPC*_ in ΔTn*3*-Variants 1a/b. Of note is that all isolates in phylogroup B1 and F harbored *bla*_*KPC*_ in ΔTn*3*-Variants, while the isolates of phylogroup C and D harbored *bla*_*KPC*_ in Tn*4401a*.

When the genetic environment of *bla*_NDM–1_ was analyzed, we found the typically conserved gene order found in previous studies (Martino et al., 2019) as follows: IS*Kpn-14*-ΔIS*Aba125*-*bla*_NDM_-*ble*_MBL_-*trpF-tat-dct-groES-groEL* (Figure 1). Moreover, *bla*_NDM_ isolates were co-localized on the same contig as the IncC replicons (13/16, 81%). The three remaining isolates also harbored IncC plasmids but were not on the same contig as *bla*_NDM_, possibly due to assembly issues. In addition, 15/16 isolates were positive for *rmtC*, *bla*_CMY–6_, and *sul1*, which are known hallmarks of *bla*_NDM_-IncC type 1 plasmids already shown to be circulating in Argentina (Martino et al., 2019). The remaining three metallo enzymes, *bla*_IMP–8_, and *bla*_VIM–1_ were found as the first cassette of class 1 integrons. Specifically, the gene order found in ECO-60 was as follows: 5′CS, *bla*_VIM–1_, *aac*(*6*′)-1b, *aph*(3′)-II, *ant*(3′)-Ia, *catB*, 3′CS. In ECO39, the gene order was 5′CS, *bla*_IMP–8_, *aadA1*, and putative reverse transcriptase. The close environment was not possible to see because *bla*_IMP–8_ was located on truncated contigs.

## Discussion

In the present work, we performed a longitudinal and retrospective study where we investigated the phenotypic and genetic characteristics of 71 carbapenemase-producing *E. coli* isolates referred to the NRLAR from 46 microbiology clinical laboratories across Argentina between 2008 and 2017. Our primary aim was to determine the distribution of carbapenemase-producing *E. coli* across the country, considering the local and global recognition of ExPEC as reservoirs and/or disseminators of clinically relevant β-lactamases. Interestingly, CC10 was the first clone in abundance followed by CC/ST131. In addition, after initial molecular characterization, we confirmed KPC-2 in most isolates (73%), followed by NDM-1 (23%), IMP-8 (3%), and VIM-1 (1%), with two isolates co-producing OXA-163 or OXA-439 and three isolates co-producing MCR-1 among other resistance genes.

Argentina has been endemic for KPC since 2010, harbored and disseminated by *K. pneumoniae* ST258, ST11, and other clonal types (Gomez et al., 2011; De Belder et al., 2018). Progressively, the detection of KPC in other non-*K. pneumoniae Enterobacterales*, such as *E. coli*, increased. Recent reports that analyzed global *E. coli* isolates found NDM as the most common carbapenemase in strains from Asia, while KPC was second with regional variations especially in North America (Peirano et al., 2014; Johnston et al., 2021). In Argentina, NDM was first detected in *Providencia rettgeri*, and although it used to be the preferred species, it has now spread mostly to *K. pneumoniae* and other *Enterobacterales* through IncC replicons, as shown here (Martino et al., 2019). In addition, two KPC-2 isolates co-produced class D β-lactamases: ECO18 (ST1844, phylogroup B1) the locally disseminated *bla*_OXA–163_, and ECO101 (ST491, phylogroup B2), *bla*_OXA–439_, a point mutation variant from *bla*_OXA–163_. OXA-163 is an acquired OXA-48-like β-lactamase mostly detected in *Enterobacterales* and whose phenotypic detection is a challenge because of their weak carbapenem hydrolysis. Since its first report in 2011, OXA-163 is now extensively spread throughout Argentina, and several variants like OXA-247 and OXA-438 have emerged. To our best knowledge, OXA-439 is still uncharacterized but was previously reported in an *E. coli* from Argentina, suggesting that this is a local variant as well (Kazmierczak et al., 2018).

Full-genome analysis enabled us to define ExPEC isolates according to virulence gene content. We found that 76% of the isolates harbored the virulence genes to be considered as ExPEC. In agreement with previous reports, phylogroups D and B2 had the highest score of virulence genes (Sarowska et al., 2019). Moreover, *fimH*, the gene involved in adhesion properties and regulation, was found in 96% of the isolates as seen in previous reports (Sarowska et al., 2019). When present, our isolates harbored virulence genes that contribute to fitness for adaptability and colonization (*e*.*g*., adhesins and siderophores among others; Supplementary Table 1) rather than to promote infection (Mokady et al., 2005). The non-ExPEC isolates harbored insufficient virulence genes to reach ExPEC criteria even though they had been obtained from infection sites outside the intestine. According to the literature, there is still uncertainty about the precise definition of factors differentiating ExPEC from commensal or infecting *E. coli* (Manges et al., 2019; Sarowska et al., 2019).

To understand the distribution of *E. coli* lineages and clones of carbapenemase-producing *E. coli* in Argentina, we analyzed the MLST distribution using Achtman MLST classification scheme and phylogenetic analysis. We detected seven of the eight *E. coli* phylogroups known to date. Phylogroup A provided most of the isolates, followed by phylogroups B2, B1, D, C, F, and E. In consequence, the major clone detected was CC10 (*n* = 16) that falls within phylogroup A and included seven STs, followed by CC131 (*n* = 10) which is grouped in phylogroup B2. In contrast to our findings, in a systematic review performed to infer the contribution of global ExPEC lineages in human infections, phylogroup B2 with ST131 was seen as the most abundant among clinical isolates in Central and South America, followed by other STs like ST69 and ST73, which, in our study, were represented only by two and one isolate, respectively (Manges et al., 2019). In addition, ST131, with or without carbapenemase production, was found to be proportionally higher in detection, followed by ST69 and third ST10 (Manges et al., 2019). Moreover, in 2014, Peirano et al. (2014) reported that carbapenemase-producing *E. coli* ST131 was the largest group of global samples studied. When analyzing ST131 subclones, we found H30Rx as the major subclone, which is in agreement with other studies (Pitout, 2012a), followed by H30R1 and H22 (Johnson et al., 2017; Johnston et al., 2021).

CC10 within phylogroup A was detected mostly in hospitals in AMBA, in contrast to other CCs that showed a heterogeneous geographical distribution. Moreover, CC10 was the main clone among MBL producers, which was detected in seven of 19 MBLs. This clone was the major MBL producer (six NDM-1 and one IMP-8). This clone has been reported, with or without carbapenemase production, in several continents, mainly in wild, companion, or food production animals, in plant-based foods, retail meats, wastewater, rivers and urban streams as well as part of the human intestinal microbiome (Nascimento et al., 2017; Reid et al., 2019).

Other relevant clones detected in this study were CC155 within phylogroup B1, detected in five isolates in AMBA and southern provinces from invasive and non-invasive sources as KPC and NDM producers. Reports from Brazil, our neighbor country, have detected MDR CC155 in wild birds and non-migratory birds in urban areas, expressing the antimicrobial resistance pattern of animal and human populations (Furlan et al., 2020). Another relevant clone found was CC38 in phylogroup D, which was a KPC producer detected in AMBA. This clone was found fifth in abundance globally, with highest prevalence in Asia than elsewhere (Manges et al., 2019). Other significant CC/STs were also detected here with fewer representatives, such as CC14/ST1193 in phylogroup B2 and CC23/ST88 and ST410 in phylogroup C. These clones are also relevant considering that they were identified within the top 20 ExPEC list of global strains mostly detected in clinical isolates or food animals (Manges et al., 2019).

This study has several limitations. Firstly, some clinical and epidemiological data were missing from databases. Secondly, our samples do not cover the full year of 2017 due to temporary operational limitations. Thirdly, our sample size, even though representative, has limited statistical analysis, and fourthly, we did not have access to long read sequencing to complete the plasmid analysis. However, short read sequencing of representative carbapenemase-producing *E. coli* isolates from Argentina allowed us to achieve a detailed characterization of the distribution of relevant *E. coli* clones.

In conclusion, in this study, we provide insight into the diversity of phylogroups, clones, subclones, serotypes, and resistance and virulence genes in clinical carbapenemase-producing *E. coli* causing extraintestinal infections. Specifically, we found CC10 to be first in abundance instead of ST131. Future studies are necessary to determine the local or regional impact of the COVID-19 pandemic on carbapenemase-producing ExPEC epidemiology.

## Data Availability Statement

The datasets presented in this study can be found in online repositories. The names of the repository/repositories and accession number(s) can be found in the article/Supplementary Material.

## “Carbapenemasas-ExPEC” Group

Sanat. Privado aconcagua (CDBA), Piersigulli A.; H. Aleman, O. Veliz; Sanat. Anchorena, N. Giudice; Htal. Britanico, A. Sujemecki; Hzga. Carlos Bocalandro, N. Cerda; Clinica Modelo Moron, Leticia Bardi; Clinica Y. Maternidad Suizo Argentina, A. Rodriguez; H. Gral. de Agudos Cosme Argerich, M. Badi; H. Gral de Agudos “C. G. Durand”, M. Flabiani; H. Evita de Lanus, A. Togneri; ICCYC, Fundacion Favaloro, P. Andrés; H. de Niños Ricardo Gutierrez, E. Biondi; H. Area Cipolletti, M. Rocallo; H. de Agudos Parmenio Piñero, M. Gerardo; H. Sanguinetti (Comodoro Rivadavia), S. Ortiz; H. Heller, H. Sauer; H. “Julio Perrando”, M. Carol Rey; H. Naval, J. C. Pidone; H. Vlla. El Libertador Ppe. de Asturias (Córdoba), Bongiovanni M. E.; H. Privado de Cordoba, M. Vilaro; H. Regional Rio Gallegos; H. Santojanni, C. Alfonodo; H. San Martin (Parana), M. Z. Bartoli; H. San Roque (CDBA) L. Vacaflor; H. Infantil Municipal de Cordoba, L. Gonzalez; H. Italiano (CABA), M. A. Visus; H. Gral de Agudos “J. A. Fernandez”, L. Errecalde; Laboratorio San Carlos, B. Baudaz; Laboratorio Virreyes, C. Tula; Instituto de Investigaciones Médicas Alfredo Lanari, Da. de Paulis A.; H. Guillermo Rawson (San Juan), A. Littvik; H. Militar Central Cortez B.; Sanatorio Mitre, Bottinelli, Cifarelli; H. Prov. de Neuq. “Castro Rendón”, M. R. Nuñez; Htall Arturo Oñativia - Rafael Calzada, B. S. A. S., Mariñansky A. L.; H. Gral de Agudos “J. A. Penna” (CABA), Romeo A. M.; H. Nac. Alejandro Posadas, A. Di Bella; H. de Agudos Ramos Mejia, Archuby D.; H. Guillermo Rawson (Cordoba), A. Littvik; Sanatorio Trinidad Mitre, Bottinelli L.; Sanatorio Güemes (Caba), S. Zarate; HIGA José San Martín de La Plata, Reynaldi M.; H. Velez Sarsfield, Manganello S.

## Author Contributions

MBS and DD: methodology, data curation, analysis, and writing the manuscript. JM, DF, CL, MR, TP, JC, and ET: methodology, data curation, and analysis. MOS, CV, and AR: methodology. FP and AC: conception, analysis, revision, and supervision. AER and SG: methodology, project supervision and administration, conception, design of the study, writing, review, and editing the manuscript. All authors contributed to manuscript revision, read, and approved the submitted version.

## Conflict of Interest

The authors declare that the research was conducted in the absence of any commercial or financial relationships that could be construed as a potential conflict of interest.

## Publisher’s Note

All claims expressed in this article are solely those of the authors and do not necessarily represent those of their affiliated organizations, or those of the publisher, the editors and the reviewers. Any product that may be evaluated in this article, or claim that may be made by its manufacturer, is not guaranteed or endorsed by the publisher.
